# Association of Composite Metabolic, Inflammatory, and Nutritional Indices with Right Ventricular Dysfunction in Acute Pulmonary Embolism: A Retrospective Cohort Study

**DOI:** 10.3390/jcdd13080348

**Published:** 2026-07-24

**Authors:** Murat Karamanlıoğlu, Pınar Akın Kabalak

**Affiliations:** 1 Department of Cardiology, Ankara Atatürk Sanatorium Training and Research Hospital, University of Health Sciences, 02690 Ankara, Türkiye; 2 Department of Chest Diseases, Ankara Atatürk Sanatorium Training and Research Hospital, University of Health Sciences, 02690 Ankara, Türkiye; pinarakinn@yahoo.com

**Keywords:** acute pulmonary embolism, right ventricular dysfunction, D-dimer-to-albumin ratio, prognostic nutritional index, C-reactive protein-to-albumin ratio, triglyceride–glucose index, risk stratification, computed tomography pulmonary angiography

## Abstract

Background/Objectives: Right ventricular dysfunction (RVD) is a major determinant of adverse outcomes in patients with acute pulmonary embolism (PE). Several laboratory-derived composite indices reflecting metabolic, inflammatory, thrombotic, and nutritional status have been proposed as prognostic markers in cardiovascular diseases; however, their relationship with imaging-defined RVD in acute PE remains insufficiently investigated. This study aimed to evaluate the association between metabolic, inflammatory, thrombotic, and nutritional indices and RVD in patients with acute PE. Methods: This retrospective observational cohort study included 270 patients with acute PE treated at a tertiary referral center between January 2024 and March 2026. Patients were categorized according to the presence or absence of RVD determined by echocardiographic and/or computed tomography pulmonary angiography criteria. The triglyceride–glucose (TyG) index, triglyceride/high-density lipoprotein cholesterol ratio (TG/HDL-C), blood urea nitrogen-to-albumin ratio (BAR), C-reactive protein-to-albumin ratio (CAR), lactate-to-albumin ratio (LAR), D-dimer-to-albumin ratio (DAR), fibrinogen-to-albumin ratio (FAR), and prognostic nutritional index (PNI) were calculated from admission laboratory data. Logistic regression, receiver operating characteristic (ROC), DeLong, and correlation analyses were performed. Results: Among the 270 patients, 118 (43.7%) had RVD and 152 (56.3%) did not. Patients with RVD demonstrated significantly higher thromboembolic burden, more severe imaging findings, and worse clinical outcomes. All investigated composite indices differed significantly between groups (all *p* < 0.001). In multivariable logistic regression analyses, TyG index (OR: 1.63, 95% CI: 1.01–2.63), BAR (OR: 1.15, 95% CI: 1.05–1.26), CAR (OR: 1.03, 95% CI: 1.01–1.06), LAR (OR: 2.04, 95% CI: 1.12–3.72), DAR (OR: 1.18, 95% CI: 1.04–1.34), and PNI (OR: 0.93, 95% CI: 0.88–0.98) remained independently associated with RVD. ROC analysis demonstrated that DAR yielded the numerically highest AUC (AUC: 0.858, 95% CI: 0.812–0.904), but was not statistically superior to CAR (AUC: 0.846), PNI (AUC: 0.837), and LAR (AUC: 0.823). Correlation analysis revealed that DAR exhibited the strongest association among the composite indices with both RV/LV ratio on computed tomography pulmonary angiography (r = 0.612, *p* < 0.001) and tricuspid annular plane systolic excursion (r = −0.551, *p* < 0.001). Conclusions: Several metabolic, inflammatory, thrombotic, and nutritional indices were associated with imaging-defined RVD in acute PE. DAR yielded the numerically highest AUC and the strongest correlations among the composite indices but was not statistically superior to CAR, PNI, or LAR. These exploratory findings do not establish incremental value beyond D-dimer, cardiac biomarkers, established risk scores, or imaging.

## 1. Introduction

Acute pulmonary embolism (PE) is a potentially life-threatening cardiovascular emergency in which pulmonary arterial obstruction abruptly increases pulmonary vascular resistance and right ventricular (RV) afterload. Short-term prognosis is largely determined by hemodynamic status and the ability of the RV to compensate for this pressure overload [1,2,3,4]. Accordingly, contemporary risk-stratification strategies incorporate clinical findings, cardiac biomarkers, and evidence of RV dysfunction (RVD) on echocardiography or computed tomography pulmonary angiography (CTPA) [5,6,7,8,9].

In addition to cardiac troponins and natriuretic peptides, several routinely available laboratory parameters reflect biological processes that may accompany severe acute PE, including coagulation activation, inflammation, tissue hypoperfusion, renal-hemodynamic stress, and impaired nutritional reserve. Composite indices such as the blood urea nitrogen-to-albumin ratio (BAR), C-reactive protein-to-albumin ratio (CAR), lactate-to-albumin ratio (LAR), D-dimer-to-albumin ratio (DAR), fibrinogen-to-albumin ratio (FAR), prognostic nutritional index (PNI), triglyceride–glucose (TyG) index, and triglyceride/high-density lipoprotein cholesterol ratio (TG/HDL-C) combine two or more of these biological domains and can be calculated from routine admission tests [10,11,12,13,14].

However, these indices are not specific to PE. Similar abnormalities have been reported in sepsis, acute heart failure, acute coronary syndromes, and other critical illnesses [15,16,17]. No established value or pattern of these indices can reliably differentiate PE from these conditions. Their interpretation in acute PE is therefore context-dependent and should follow objective confirmation of PE. Whereas their elevation in sepsis may predominantly reflect infection-related systemic inflammation and distributive hypoperfusion, and in acute coronary syndromes it may accompany myocardial ischemia and metabolic stress, in confirmed acute PE these indices may reflect the combined effects of fibrin turnover and thrombotic burden, acute RV pressure overload, systemic hypoperfusion, inflammation, and reduced physiological reserve. Thus, they should be considered potential adjunctive risk markers rather than disease-specific diagnostic biomarkers.

Most previous studies have evaluated individual indices primarily in relation to mortality or overall, PE severity, while their comparative associations with imaging-defined RVD remain insufficiently characterized. Therefore, this study aimed to evaluate the associations of admission-based metabolic, inflammatory, thrombotic, and nutritional indices with RVD in patients with confirmed acute PE and to compare their discriminatory performance within this population.

## 2. Materials and Methods

### 2.1. Study Design and Patient Selection

This single-center retrospective observational cohort study was conducted at Ankara Atatürk Sanatorium Training and Research Hospital, a tertiary referral center in Ankara, Türkiye. All consecutive adult patients aged ≥ 18 years with objectively confirmed acute PE diagnosed between 1 January 2024 and 31 March 2026 were screened through the hospital electronic medical records system. Patients diagnosed in the emergency department, inpatient wards, or intensive care unit were eligible. Patients who developed PE during hospitalization for another primary medical or surgical condition were also included. No sampling or selection was performed according to hemodynamic status, PE severity, RV findings, or laboratory results.

PE was confirmed by CTPA or, when CTPA was contraindicated or non-diagnostic, by ventilation–perfusion scintigraphy in conjunction with compatible clinical findings. The study protocol was approved by the Scientific Research Ethics Committee of Ankara Atatürk Sanatorium Training and Research Hospital (Approval No. 2024-BÇEK/556; 6 May 2026) and was conducted in accordance with the Declaration of Helsinki.

Patients were eligible for inclusion if they were aged 18 years or older, had a confirmed diagnosis of acute pulmonary embolism established by computed tomography pulmonary angiography (CTPA), ventilation–perfusion scintigraphy, or compatible clinical and radiological findings, and had available laboratory data sufficient for calculation of the investigated metabolic, inflammatory, and nutritional indices. In addition, patients were required to have an assessment of right ventricular (RV) function performed by echocardiography and/or CTPA. Patients were excluded if they were younger than 18 years, had chronic thromboembolic pulmonary hypertension, or lacked a definitive diagnosis of acute pulmonary embolism. Additional exclusion criteria included advanced structural heart disease, severe valvular heart disease, or chronic severe right heart failure that could independently influence RV function; missing laboratory data preventing calculation of the study indices; duplicate records resulting from repeated hospital admissions; major trauma or cardiopulmonary resuscitation prior to admission that could substantially affect biochemical parameters; and pregnancy. For patients with multiple admissions during the study period, only the first eligible hospitalization was included in the analysis to avoid duplication of data. Active malignancy was defined as a solid or hematological malignancy diagnosed or treated within the preceding six months or the presence of recurrent, locally advanced, or metastatic disease. A remote history of cancer without current treatment or active disease was not classified as active malignancy.

### 2.2. PE Presentation Setting, Clinical Risk, and Echocardiography Protocol

Patients were classified according to the setting of PE presentation. Out-of-hospital PE was defined as PE that was the primary reason for hospital presentation or was identified during the initial emergency evaluation. In-hospital PE was defined as PE diagnosed after admission for another primary medical or surgical condition.

High-risk PE was defined by hemodynamic instability at presentation, including cardiac arrest, obstructive shock requiring vasopressor support to maintain systolic blood pressure ≥ 90 mmHg together with evidence of end-organ hypoperfusion, or persistent hypotension defined as systolic blood pressure < 90 mmHg or a decrease of ≥40 mmHg for more than 15 min that was not attributable to arrhythmia, hypovolemia, or sepsis [2].

According to the institutional clinical approach, bedside transthoracic echocardiography was performed immediately in patients with hemodynamic instability or suspected high-risk PE. In hemodynamically stable patients, echocardiography was requested as part of the initial risk assessment and was targeted within 24 h after objective confirmation of PE. For patients with more than one examination, the first echocardiogram obtained during the index PE episode was included. Echocardiography was available in 238 patients (88.1%). The median interval between objective confirmation of PE and the first echocardiographic examination was 10 (IQR: 5–20) hours, and 198 of 238 examinations (83.2%) were performed within 24 h.

### 2.3. Definition of Right Ventricular Dysfunction

The primary study outcome was imaging-defined RVD. RVD was defined according to previously established imaging criteria in acute pulmonary embolism [2,18,19]. RVD status was independently re-adjudicated using the first echocardiographic and/or CTPA examination obtained during the index PE episode. Patients were classified as having RVD if at least one of the following criteria was present: RV/LV ratio > 1.0 on echocardiography, TAPSE < 16 mm, RV dilatation or hypokinesia on echocardiography, RV/LV ratio ≥ 1.0 on CTPA, or explicit documentation of acute RV strain or dysfunction in the final imaging report. Patients without any of these findings were classified as not having RVD. Two investigators independently reviewed the imaging data, and disagreements were resolved by consensus.

### 2.4. Data Collection and Sample Size

Data were collected retrospectively from the hospital electronic medical records system and included demographic, clinical, imaging, laboratory, treatment, and outcome variables. Demographic characteristics comprised age and sex. Clinical data included presenting symptoms, systolic and diastolic blood pressure, heart rate, respiratory rate, oxygen saturation, comorbid conditions such as hypertension, diabetes mellitus, chronic obstructive pulmonary disease, coronary artery disease, chronic kidney disease, and malignancy, as well as the need for intensive care unit admission, mechanical ventilation, vasopressor support, thrombolytic therapy, length of hospital stay, length of intensive care unit stay, and in-hospital mortality.

The CTPA and echocardiographic variables used to determine RVD were extracted from the first examinations obtained during the index PE episode and are described in Section 2.3. Laboratory parameters recorded at admission included white blood cell, neutrophil, lymphocyte, and platelet counts; serum glucose, blood urea nitrogen (BUN), creatinine, albumin, C-reactive protein (CRP), lactate, D-dimer, fibrinogen, triglycerides, and high-density lipoprotein cholesterol (HDL-C) levels; and cardiac troponin and B-type natriuretic peptide (BNP) or N-terminal pro-BNP (NT-proBNP) levels when available.

To evaluate the metabolic, inflammatory, thrombotic, and nutritional status of the study population, several composite indices were calculated using admission laboratory parameters. The triglyceride–glucose (TyG) index was calculated as ln [triglyceride (mg/dL) × glucose (mg/dL)/2], while the triglyceride-to-HDL cholesterol ratio (TG/HDL-C) was calculated by dividing triglyceride concentration by HDL-C concentration. The blood urea nitrogen-to-albumin ratio (BAR), C-reactive protein-to-albumin ratio (CAR), lactate-to-albumin ratio (LAR), D-dimer-to-albumin ratio (DAR), and fibrinogen-to-albumin ratio (FAR) were calculated by dividing the respective biomarker levels by serum albumin concentration. The prognostic nutritional index (PNI) was calculated using the formula: PNI = [10 × albumin (g/dL)] + [0.005 × total lymphocyte count (/mm^3^)].

A priori sample size analysis was performed using G*Power software (version 3.1.9.7; Heinrich Heine University, Düsseldorf, Germany). Because of the retrospective design, all consecutive eligible patients were included. An a priori power analysis indicated that a minimum sample size of 266 patients was required, whereas 270 patients were ultimately included.

### 2.5. Statistical Analysis

All statistical analyses were performed using IBM SPSS Statistics version 27.0 (IBM Corp., Armonk, NY, USA) and R version 4.3.0 (R Foundation for Statistical Computing, Vienna, Austria). Continuous variables were assessed for normality using the Kolmogorov–Smirnov and Shapiro–Wilk tests. Normally distributed variables are presented as mean ± standard deviation, non-normally distributed variables as median (interquartile range), and categorical variables as number (%). Between-group comparisons were performed using Student’s *t*-test, the Mann–Whitney U test, the chi-square test, or Fisher’s exact test, as appropriate. Univariable logistic regression was used to evaluate factors associated with RVD. Based on clinical relevance and univariable findings, the primary multivariable model included age, active malignancy, heart rate, oxygen saturation, main pulmonary artery involvement, saddle embolism, troponin, BNP/NT-proBNP, and the investigated composite indices. Results are reported as ORs with 95% CIs. Multicollinearity was assessed using variance inflation factors. ROC analysis was used to assess the ability of each index to discriminate between patients with and without RVD. AUCs, Youden index-derived cutoffs, sensitivity, and specificity were calculated, and selected AUCs were compared using the DeLong method. Because no adjustment for multiple comparisons was applied, pairwise ROC comparisons were considered exploratory. Associations between laboratory indices and quantitative imaging parameters were evaluated using Spearman correlation analysis. Post hoc sensitivity analyses were performed after excluding patients with high-risk PE and after stratification by out-of-hospital versus in-hospital PE. In the stratified analyses, each composite index was evaluated in a separate model adjusted for the predefined clinical covariates. Index-by-presentation-setting interaction terms were used to examine heterogeneity between the two settings. In an additional model, DAR was replaced by log_2_-transformed D-dimer; thus, its OR represents the change in the odds of RVD associated with each doubling of D-dimer. DAR and D-dimer were not included simultaneously because of their mathematical dependence. All tests were two-sided, and *p* < 0.05 was considered statistically significant. Predefined clinical covariates were age, active malignancy, heart rate, oxygen saturation, main pulmonary artery involvement, saddle embolism, cardiac troponin, and natriuretic peptide level. Missing values were not imputed; each analysis was based on complete cases for the variables included in the corresponding model. Because BNP and NT-proBNP have different analytical ranges, their raw concentrations were not pooled directly. Each value was normalized to the assay-specific upper reference limit and subsequently log_2_-transformed. Accordingly, the reported OR represents the change in the odds of RVD associated with each doubling of the normalized natriuretic peptide level. All statistical tests were two-sided, and a *p*-value < 0.05 was considered statistically significant.

## 3. Results

**Figure 1:**
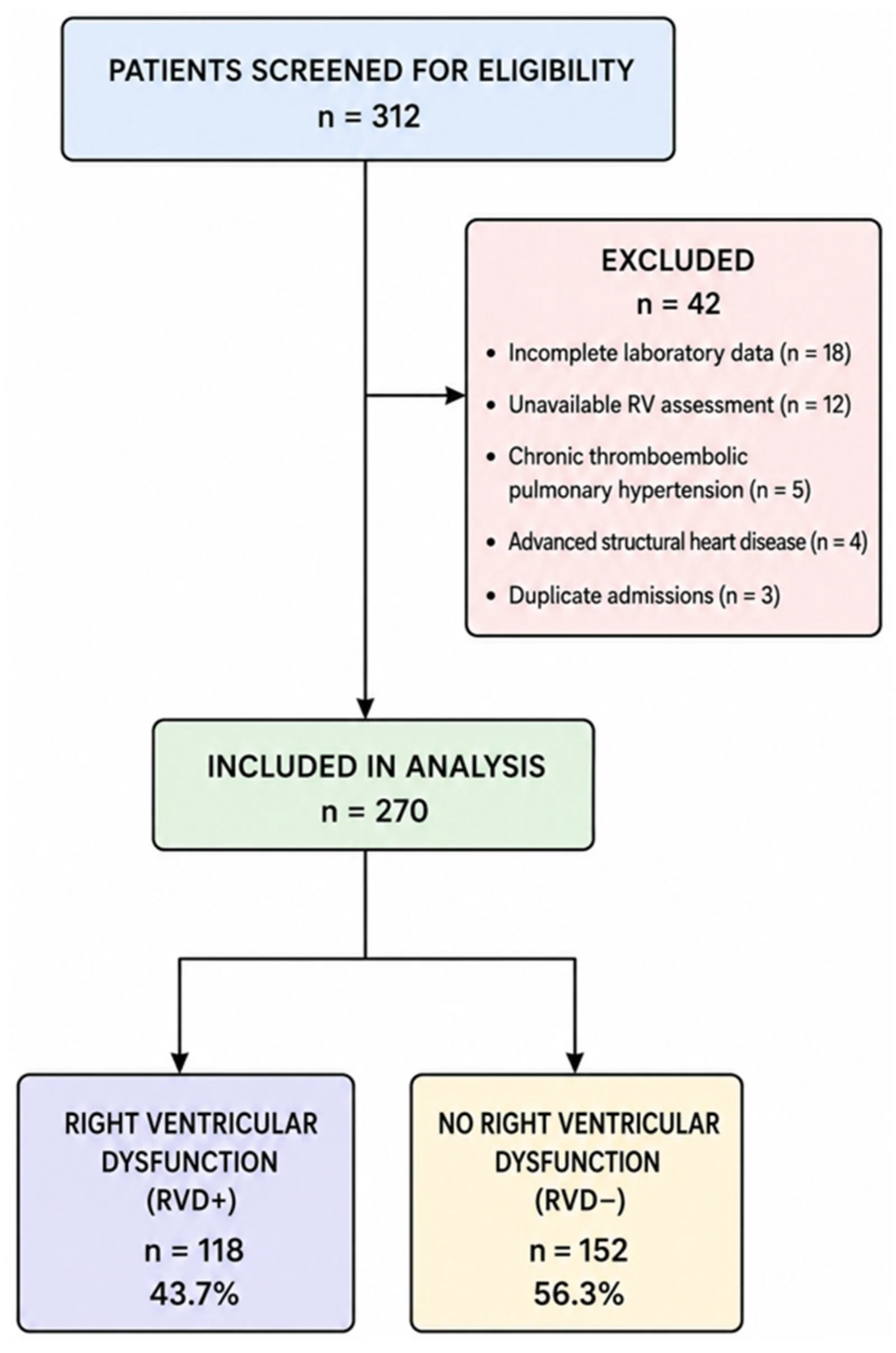
Flowchart of the study.

A total of 312 patients with PE were screened for eligibility during the study period. After exclusion of patients with incomplete laboratory data (n = 18), unavailable right ventricular assessment (n = 12), chronic thromboembolic pulmonary hypertension (n = 5), advanced structural heart disease (n = 4), and duplicate admissions (n = 3), 270 patients were included in the final analysis. Among the included patients, 118 (43.7%) were classified as having RVD, whereas 152 (56.3%) had no evidence of RVD based on echocardiographic and/or computed tomography criteria (Figure 1).

The baseline demographic and clinical characteristics of the study population are shown in Table 1. Patients with RVD were older and more frequently had active malignancy. They also presented with greater tachycardia, hypoxemia, hemodynamic compromise, and higher requirements for intensive care and organ support. Other baseline comorbidities were broadly comparable between the groups (Table 1).

Echocardiography was available more frequently in patients with RVD than in those without RVD. Patients with RVD also had a greater anatomical thromboembolic burden, with more frequent bilateral PE, main pulmonary artery involvement, and saddle embolism, whereas isolated segmental or subsegmental involvement was less frequent. Lobar artery involvement did not differ significantly between the groups (Table 2). Imaging parameters used directly for RVD adjudication were not subjected to inferential between-group comparisons.

Admission laboratory findings and calculated composite indices are shown in Table 3. Admission inflammatory, thrombotic, metabolic, and cardiac biomarkers differed significantly according to RVD status. The RVD group had higher TyG, TG/HDL-C, BAR, CAR, LAR, DAR, and FAR values and lower PNI values (Table 3).

Univariable and multivariable logistic regression analyses are shown in Table 4. Univariable analyses identified significant associations between RVD and age, active malignancy, heart rate, oxygen saturation, main pulmonary artery involvement, saddle embolism, cardiac biomarkers, and all investigated composite indices. In the multivariable model, heart rate, oxygen saturation, main pulmonary artery involvement, troponin, BNP/NT-proBNP, TyG, BAR, CAR, LAR, DAR, and PNI remained associated with RVD, whereas age, active malignancy, saddle embolism, TG/HDL-C, and FAR did not. No concerning multicollinearity was detected among the composite indices (all reported VIFs < 2.0) (Table 4).

After excluding 39 patients with high-risk PE, 231 patients remained in the sensitivity cohort, of whom 89 (38.5%) had RVD. In the adjusted analyses, BAR, CAR, LAR, and DAR remained positively associated with RVD, whereas higher PNI remained inversely associated with RVD. In contrast, the associations of the TyG index, TG/HDL-C ratio, and FAR were attenuated and were no longer statistically significant (Supplementary Table S1). Of the 270 patients, 192 (71.1%) had out-of-hospital PE and 78 (28.9%) had in-hospital PE. RVD was identified in 74 patients (38.5%) with out-of-hospital PE and 44 patients (56.4%) with in-hospital PE. In the adjusted stratified analyses, CAR, DAR, and PNI remained significantly associated with RVD in both presentation settings. BAR and LAR remained significant only in the out-of-hospital subgroup, whereas their associations were attenuated in patients with in-hospital PE. Although the TyG index was associated with RVD in the out-of-hospital subgroup, TG/HDL-C and FAR were not significant in either subgroup. No statistically significant index-by-presentation-setting interactions were observed, suggesting no clear evidence that the associations differed according to PE presentation setting (all interaction *p*-values > 0.05) (Supplementary Table S2).

In the alternative multivariable model in which DAR was replaced by log_2_-transformed D-dimer, increasing D-dimer remained independently associated with RVD. Each doubling of D-dimer was associated with a 43% increase in the adjusted odds of RVD (adjusted OR: 1.43, 95% CI: 1.15–1.78; *p* = 0.001) (Supplementary Table S3).

**Figure 2:**
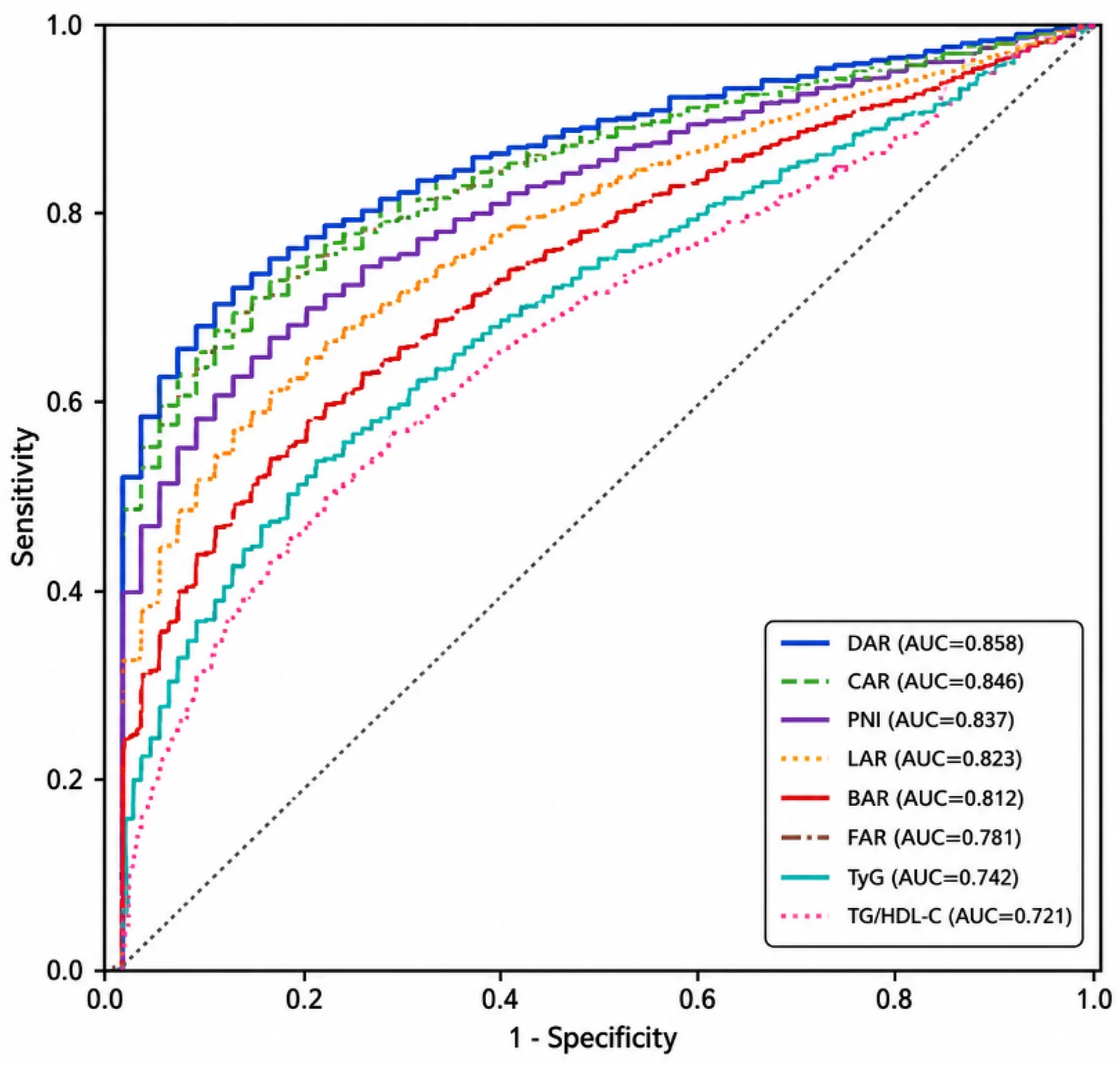
ROC curves of the investigated composite indices for prediction of RVD.

ROC analysis showed that all investigated composite indices significantly discriminated between patients with and without RVD (all AUC *p* < 0.001). DAR yielded the numerically highest AUC (0.858, 95% CI: 0.812–0.904), followed by CAR, PNI, and LAR, whereas the metabolic indices showed lower discrimination (Table 5 and Figure 2).

Pairwise comparisons of ROC curves using DeLong analysis are shown in Table 6. DeLong analysis showed no significant differences between DAR and CAR, PNI, or LAR. In unadjusted pairwise comparisons, DAR, CAR, and PNI had nominally higher AUCs than several lower-performing indices, particularly FAR, TyG, and TG/HDL-C. Because no adjustment for multiple comparisons was applied, these findings should be considered exploratory (Table 6).

**Figure 3:**
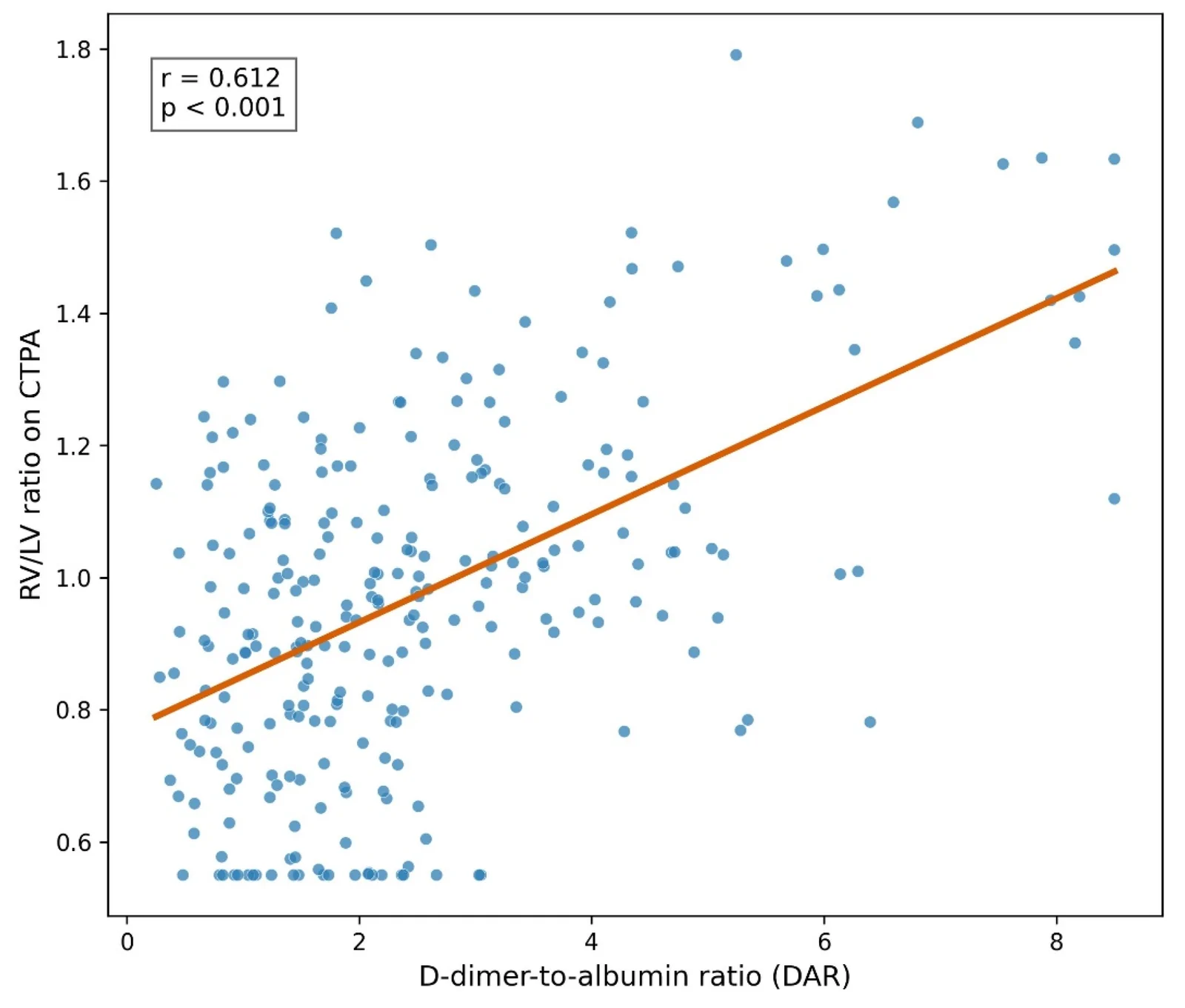
Scatter plot demonstrating the relationship between the DAR and RV/LV ratio.

**Figure 4:**
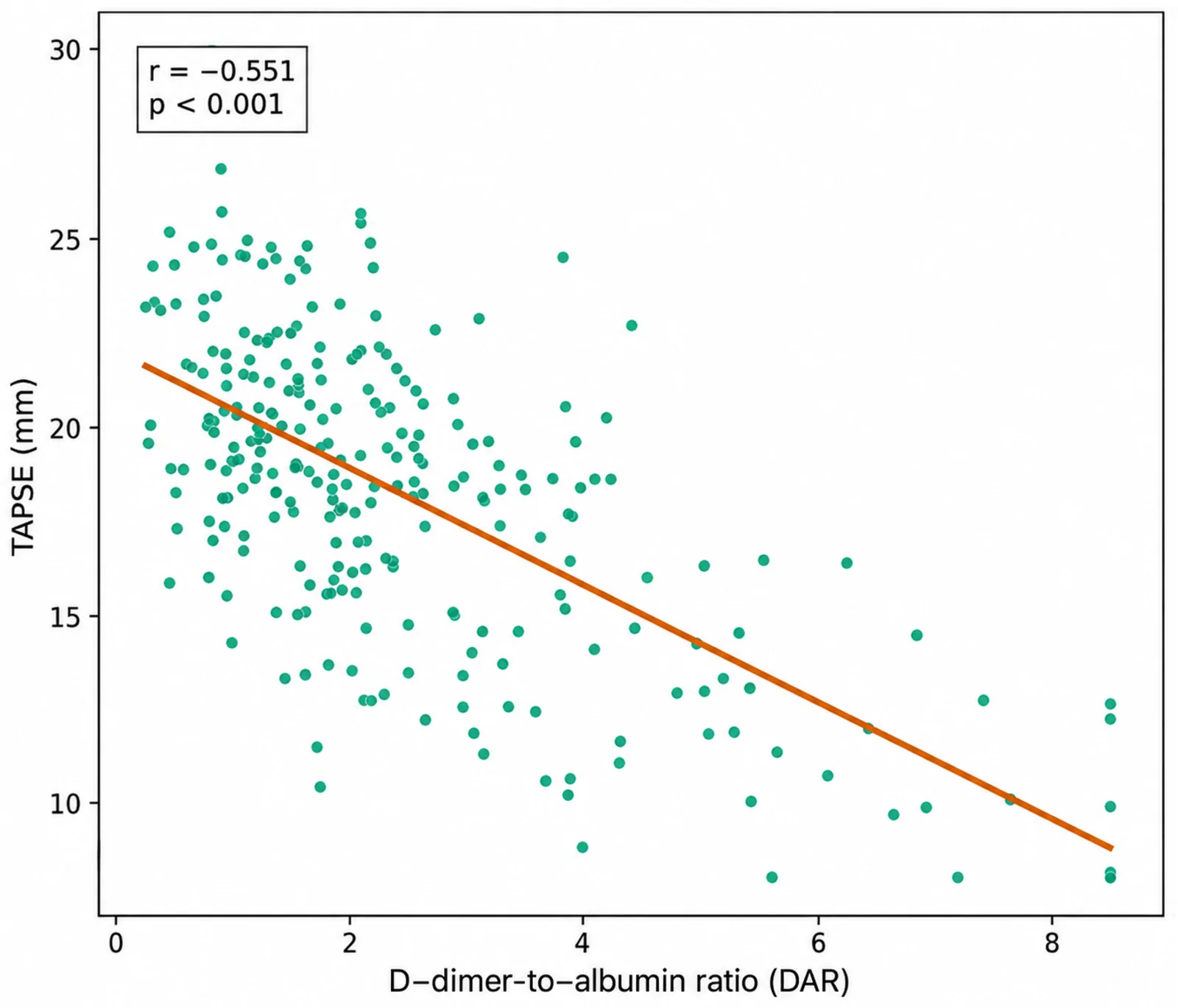
Scatter plot demonstrating the relationship between the DAR and TAPSE.

Correlation analysis between composite indices and markers of RVD are shown in Table 7. Correlation analyses showed that all composite indices were significantly correlated with RV/LV ratio on CTPA and echocardiography and with TAPSE (all *p* ≤ 0.002). Among the composite indices, DAR yielded the numerically largest absolute correlation coefficients with CTPA-derived RV/LV ratio (r = 0.612), echocardiographic RV/LV ratio (r = 0.587), and TAPSE (r = −0.551). BNP/NT-proBNP showed the strongest overall correlations among the conventional biomarkers, while troponin was also significantly correlated with all imaging parameters (Table 7, Figure 3 and Figure 4).

Management and clinical outcomes according to RVD status are shown in Table 8. Patients with RVD more frequently required intensive care, ventilatory and vasopressor support, thrombolytic therapy, and catheter-directed intervention or embolectomy and had longer ICU and hospital stays. Hemodynamic deterioration, cardiopulmonary arrest, major bleeding, deep vein thrombosis, and mortality were also more frequent in the RVD group. In-hospital mortality was 17.8% versus 3.9%, and 30-day mortality was 20.3% versus 4.6% (both *p* < 0.001). Recurrent PE did not differ significantly between the groups. These unadjusted comparisons should be interpreted descriptively (Table 8).

## 4. Discussion

In this retrospective cohort study of patients with acute PE, we examined the associations between index-episode metabolic, inflammatory, thrombotic, and nutritional indices and imaging-defined RVD. Patients with RVD had more extensive pulmonary arterial involvement and more frequent adverse short-term outcomes, although the outcome comparisons were unadjusted. All investigated indices were associated with RVD in univariable analyses; after multivariable adjustment, TyG, BAR, CAR, LAR, DAR, and PNI remained associated with RVD. DAR yielded the numerically highest AUC, but its discriminatory performance did not differ significantly from that of CAR, PNI, or LAR. DAR also yielded the numerically largest absolute correlation coefficients among the composite indices, whereas BNP/NT-proBNP yielded the largest coefficients among conventional biomarkers; these differences were not formally compared. Overall, these findings demonstrate associations with imaging-defined RVD but do not establish causality, biomarker superiority, or incremental clinical value beyond established PE risk-stratification strategies.

RVD is an established determinant of early clinical deterioration in acute PE. In our cohort, RVD was associated with worse short-term outcomes, while BNP/NT-proBNP showed the strongest correlations among conventional biomarkers with quantitative measures of RV function. Previous studies have evaluated different populations and outcomes. Gutte et al. assessed BNP, pro-ANP, and D-dimer for identifying imaging-detected RVD [20], whereas Barco et al. examined the prognostic implications of RVD and cardiac biomarker elevation among patients initially classified as having low-risk PE [21]. Hu et al. evaluated the association between CTPA-based markers of RV dysfunction and 30-day adverse outcomes [22]. In a large multicenter RIETE registry analysis, Siniscalchi et al. reported lower adjusted odds of 30-day all-cause and PE-related mortality among patients receiving statins at baseline [23]. However, that study evaluated treatment exposure and mortality, and its observational design does not establish a causal protective effect of statins. Unlike the low-risk population evaluated by Barco et al. [21], our cohort included patients across a broader severity spectrum, including high-risk and in-hospital PE, and used imaging-defined RVD rather than mortality as the primary outcome. Because RV/LV ratio, TAPSE, RV dilatation, and RV hypokinesia contributed directly to RVD adjudication, these variables were not subjected to inferential comparisons between the RVD groups. Accordingly, our findings should be interpreted as associations with RVD rather than evidence of a novel or comprehensive prognostic model.

Acute PE is not exclusively a consequence of mechanical pulmonary arterial obstruction but also involves a bidirectional interaction between coagulation and inflammation. Tissue factor-dependent thrombin generation promotes fibrin formation and platelet activation, while thrombin-mediated signaling can activate endothelial cells and leukocytes, increase adhesion molecule expression, and amplify inflammatory and procoagulant responses. Endothelial dysfunction, reduced thrombomodulin–protein C activity, and platelet–leukocyte interactions may further shift the hemostatic balance toward thrombosis. Siniscalchi et al. reviewed these interconnected pathways, including tissue factor expression, thrombin generation, thrombomodulin, the protein C system, and platelet activation [24]. In acute PE, D-dimer reflects the degradation of cross-linked fibrin and therefore ongoing fibrin turnover, whereas albumin may decrease in association with systemic inflammation, oxidative stress, and increased vascular permeability. Consequently, DAR may increase when thrombotic and inflammatory processes coexist. These mechanisms may also augment pulmonary vascular resistance and RV afterload beyond the effects of anatomical obstruction alone. Nevertheless, this mechanistic interpretation remains hypothesis-generating because DAR is nonspecific and its observed performance was driven, at least partly, by its D-dimer component.

Among the investigated composite indices, DAR yielded the numerically highest AUC and showed strong correlations with RV/LV ratio and TAPSE; however, its discriminatory performance was not statistically superior to that of CAR, PNI, or LAR. DAR may reflect both coagulation activation and albumin-related systemic reserve, but D-dimer itself remained independently associated with RVD when DAR was replaced by log_2_-transformed D-dimer. Thus, the observed performance of DAR appears to be driven, at least partly, by its D-dimer component. Previous studies also differed in their populations and outcomes: Gutte et al. assessed D-dimer in relation to imaging-detected RVD, Türedi et al. examined radiological PE severity, Özcan et al. and Artac et al. evaluated CAR primarily in relation to mortality, and Najarro et al. investigated CRP in relation to RVD and mortality [20,25,26,27,28]. In contrast, our primary outcome was imaging-defined RVD. Therefore, these findings indicate an association with RV compromise but do not establish causality, superiority, or incremental clinical value beyond D-dimer, cardiac biomarkers, and imaging.

BAR, LAR, and PNI were also associated with RVD, although the magnitude and precision of these associations varied across the primary and sensitivity analyses. Fang et al. evaluated BAR in a database-derived cohort restricted to critically ill patients with acute PE and used ICU mortality as the outcome [29]. Hayıroğlu et al. examined PNI in relation to in-hospital survival rather than imaging-defined RVD [30]. Evidence regarding LAR in PE remains limited and has largely been extrapolated from heterogeneous critically ill populations [14,16,31]. These differences in patient selection and outcomes limit direct comparisons with our broader tertiary-care cohort. Therefore, the present findings suggest that renal-hemodynamic stress, hypoperfusion, and immune-nutritional status may accompany RVD, but they do not establish these indices as clinically validated predictors.

The metabolic indices showed weaker associations with RVD than the leading albumin-based indices. Bilgin et al. evaluated TyG primarily in relation to mortality in pulmonary thromboembolism, while their study in ST-elevation myocardial infarction examined a different arterial thrombotic population and outcome [10,15]. In the present study, the TyG index showed only moderate discrimination, and its association was attenuated in the sensitivity analysis restricted to non-high-risk PE. Therefore, metabolic dysfunction may accompany a more severe clinical profile, but its specific contribution to RVD cannot be determined from the present retrospective data.

Albumin-based indices generally yielded numerically higher AUC values than the metabolic indices. However, DeLong analyses demonstrated no statistically significant differences among DAR, CAR, PNI, and LAR. Because these indices share albumin as a component and reflect overlapping aspects of thrombotic burden, inflammation, hypoperfusion, and physiological reserve, their similar performance may partly result from both shared pathophysiological information and mathematical coupling. Although DAR yielded the numerically highest AUC and showed strong correlations with imaging markers, the present findings do not establish its superiority or justify prioritizing it over the other leading composite indices. Therefore, these comparative results should be considered exploratory.

Current risk stratification in acute PE begins with the identification of hemodynamic instability, which defines high-risk PE and indicates the need for urgent reperfusion assessment. In hemodynamically stable patients, the ESC strategy integrates clinical risk, commonly assessed using PESI or sPESI, with imaging evidence of RVD and cardiac troponin elevation [2,20,21,32]. Patients with both RVD and elevated troponin are categorized as intermediate–high risk, whereas patients with only one or neither abnormality are generally classified as intermediate–low risk, depending on their clinical risk profile. The Bova score provides an alternative approach for staging normotensive patients using heart rate, systolic blood pressure, cardiac troponin, and RVD to estimate short-term PE-related complications [33]. Unlike these validated strategies, the indices evaluated in our study are nonspecific and were examined primarily in relation to imaging-defined RVD rather than 30-day mortality or PE-related clinical deterioration. Therefore, their AUC values cannot be directly compared with those of sPESI or the Bova score. These indices should not replace ESC risk categories, clinical scores, cardiac biomarkers, or imaging and should not currently guide reperfusion, monitoring, or discharge decisions. Their potential role, if externally validated, would be as adjunctive markers within an established risk category. Although several indices remained associated with RVD after adjustment for troponin and BNP/NT-proBNP, statistical significance in a multivariable model does not demonstrate incremental prognostic value. We did not compare nested models consisting of established clinical scores, cardiac biomarkers, and imaging findings with and without the composite indices. Moreover, because imaging-defined RVD was the primary outcome, the additive value of these indices beyond RVD itself for predicting clinical deterioration or mortality could not be assessed. Future prospective studies should use clinically relevant outcomes and formally evaluate changes in discrimination, calibration, and risk reclassification after adding these indices to established models. Prospective studies must determine whether they provide incremental value beyond the combined clinical, biomarker, and imaging approach.

Compared with previously published studies, the present study differs in several important aspects. Most earlier investigations primarily focused on mortality, overall disease severity, or individual biomarkers in patients with acute pulmonary embolism. In contrast, our study specifically evaluated imaging-defined right ventricular dysfunction as the primary outcome and simultaneously assessed multiple metabolic, inflammatory, thrombotic, and nutritional indices within the same patient cohort. Furthermore, while previous studies generally investigated CAR, BAR, PNI, or D-dimer separately, the present study directly compared the discriminatory performance for imaging-defined RVD of TyG index, TG/HDL-C ratio, BAR, CAR, LAR, DAR, FAR, and PNI using logistic regression, ROC, DeLong, and correlation analyses. To our knowledge, this is one of the first studies to comparatively evaluate these composite indices in relation to imaging-defined RVD; however, the comparative findings should be considered exploratory, and the numerically higher performance of DAR should not be interpreted as evidence of superiority over either D-dimer alone or the other leading composite indices.

This study has some limitations. First, its retrospective single-center design may limit generalizability and introduce selection bias. Second, laboratory measurements were obtained from routine clinical records, and the timing of blood sampling relative to symptom onset may have varied among patients. Third, echocardiography was not available for all patients, although RVD was assessed using echocardiography and/or CTPA according to available clinical records. Fourth, because several composite indices share albumin as a denominator or component, potential multicollinearity may be a concern; however, VIF analysis showed no significant multicollinearity among the included indices. Fifth, the study focused on associations with RVD and clinical outcomes, and causal relationships cannot be inferred. In addition, although cardiac troponin and BNP/NT-proBNP were included in the multivariable analyses, the study did not formally evaluate the incremental value of the composite indices beyond established clinical scores, cardiac biomarkers, and imaging. No nested model comparisons, changes in AUC, calibration analyses, or reclassification measures were performed. Furthermore, imaging-defined RVD was the primary outcome rather than a prospective clinical endpoint. Therefore, the present findings cannot establish whether these indices improve prediction of hemodynamic deterioration or mortality beyond currently validated risk-stratification strategies. Finally, external validation in larger, prospective, multicenter cohorts is required before these indices can be incorporated into routine clinical risk-stratification algorithms.

## 5. Conclusions

In conclusion, metabolic, inflammatory, thrombotic, and nutritional indices were significantly associated with right ventricular dysfunction in patients with acute pulmonary embolism. Patients with RVD exhibited a greater thromboembolic burden, more severe imaging findings, and worse short-term clinical outcomes than those without RVD. Among the investigated composite indices, the D-dimer-to-albumin ratio yielded the numerically highest AUC and showed strong correlations with imaging markers of right ventricular dysfunction; however, its discriminatory performance was not statistically superior to that of CAR, PNI, or LAR. Several indices remained independently associated with RVD after multivariable adjustment; however, their incremental value beyond ESC risk categories, validated clinical scores, cardiac biomarkers, and imaging was not formally evaluated. Accordingly, these indices should currently be regarded as exploratory adjunctive markers rather than validated tools for clinical decision-making. Nevertheless, prospective multicenter studies are required to validate these findings and determine their potential role in routine clinical practice.

## Figures and Tables

**Table 1:** Baseline demographic and clinical characteristics of patients with and without RVD.

Variable	Total (n = 270)	RVD (+) (n = 118)	RVD (−) (n = 152)	*p*-Value
Age, years	68.2 ± 14.1	71.4 ± 13.2	65.7 ± 14.4	0.002
Male sex, n (%)	134 (49.6)	62 (52.5)	72 (47.4)	0.410
Body mass index, kg/m^2^	28.6 ± 5.1	29.1 ± 5.3	28.2 ± 4.9	0.180
Hypertension, n (%)	138 (51.1)	67 (56.8)	71 (46.7)	0.102
Diabetes mellitus, n (%)	82 (30.4)	39 (33.1)	43 (28.3)	0.401
Coronary artery disease, n (%)	59 (21.9)	30 (25.4)	29 (19.1)	0.218
Chronic kidney disease, n (%)	37 (13.7)	21 (17.8)	16 (10.5)	0.089
Chronic obstructive pulmonary disease, n (%)	48 (17.8)	25 (21.2)	23 (15.1)	0.201
Active malignancy, n (%)	52 (19.3)	30 (25.4)	22 (14.5)	0.025
Previous DVT/PE history, n (%)	31 (11.5)	15 (12.7)	16 (10.5)	0.580
Smoking history, n (%)	93 (34.4)	44 (37.3)	49 (32.2)	0.386
Heart rate, beats/min	104 ± 22	112 ± 24	98 ± 18	<0.001
Systolic blood pressure, mmHg	118 ± 23	111 ± 24	123 ± 20	<0.001
Respiratory rate, breaths/min	24 ± 6	27 ± 6	22 ± 5	<0.001
Oxygen saturation, %	90 ± 7	86 ± 8	93 ± 5	<0.001
Shock/hypotension, n (%)	39 (14.4)	29 (24.6)	10 (6.6)	<0.001

Data are presented as mean ± standard deviation, median (interquartile range), or number (%), as appropriate. RVD: right ventricular dysfunction; ICU: intensive care unit; DVT: deep vein thrombosis; PE: pulmonary embolism.

**Table 2:** Pulmonary embolic distribution and imaging availability according to RVD status.

Variable	Total (n = 270)	RVD (+) (n = 118)	RVD (−) (n = 152)	*p*-Value
Imaging availability				
Echocardiography performed, n (%)	238 (88.1)	112 (94.9)	126 (82.9)	0.004
Pulmonary embolic distribution on CTPA				
Bilateral pulmonary embolism, n (%)	171 (63.3)	89 (75.4)	82 (53.9)	<0.001
Main pulmonary artery involvement, n (%)	86 (31.9)	58 (49.2)	28 (18.4)	<0.001
Lobar artery involvement, n (%)	143 (53.0)	67 (56.8)	76 (50.0)	0.272
Isolated segmental/subsegmental involvement, n (%)	41 (15.2)	8 (6.8)	33 (21.7)	0.001
Saddle pulmonary embolism, n (%)	38 (14.1)	29 (24.6)	9 (5.9)	<0.001

Data are presented as number (%), with percentages calculated within each column. Pulmonary embolic distribution categories may overlap, except for isolated segmental/subsegmental involvement. Between-group comparisons were performed using the chi-square test or Fisher’s exact test, as appropriate. Imaging findings used directly to define or adjudicate RVD—including RV/LV ratio, RV dilatation, RV hypokinesia, TAPSE, septal deviation, and McConnell’s sign—were excluded from inferential between-group comparisons to avoid circularity. CTPA: computed tomography pulmonary angiography; PE: pulmonary embolism; RVD: right ventricular dysfunction; RV: right ventricle; LV: left ventricle; TAPSE: tricuspid annular plane systolic excursion.

**Table 3:** Admission laboratory parameters and calculated composite indices according to RVD status.

Variable	Total (n = 270)	RVD (+) (n = 118)	RVD (−) (n = 152)	*p*-Value
White blood cell count (×10^3^/µL)	10.8 ± 4.1	12.1 ± 4.5	9.8 ± 3.4	<0.001
Neutrophil count (×10^3^/µL)	7.9 ± 3.8	9.3 ± 4.1	6.8 ± 2.9	<0.001
Lymphocyte count (×10^3^/µL)	1.52 ± 0.71	1.28 ± 0.60	1.71 ± 0.74	<0.001
Hemoglobin (g/dL)	12.7 ± 2.1	12.4 ± 2.2	12.9 ± 2.0	0.087
Platelet count (×10^3^/µL)	242 ± 89	235 ± 92	248 ± 86	0.241
Glucose (mg/dL)	138 ± 54	154 ± 61	126 ± 43	<0.001
Blood urea nitrogen (mg/dL)	31.4 ± 17.8	39.8 ± 20.1	24.9 ± 11.6	<0.001
Creatinine (mg/dL)	1.16 ± 0.54	1.24 ± 0.61	1.09 ± 0.47	0.031
Albumin (g/dL)	3.62 ± 0.58	3.28 ± 0.51	3.89 ± 0.47	<0.001
CRP (mg/L)	58 (21–118)	92 (45–161)	33 (14–72)	<0.001
Lactate (mmol/L)	2.3 (1.5–3.8)	3.4 (2.1–5.2)	1.8 (1.2–2.7)	<0.001
D-dimer (µg/mL)	5.8 (2.9–12.4)	9.8 (5.4–18.3)	3.7 (2.1–7.5)	<0.001
Fibrinogen (mg/dL)	462 ± 136	512 ± 142	423 ± 119	<0.001
Triglycerides (mg/dL)	157 ± 71	178 ± 78	141 ± 60	<0.001
HDL cholesterol (mg/dL)	40 ± 12	36 ± 10	43 ± 12	<0.001
Troponin (ng/L)	78 (24–236)	168 (72–426)	31 (14–82)	<0.001
BNP/NT-proBNP (pg/mL)	615 (218–1682)	1348 (562–2894)	296 (121–811)	<0.001
TyG index	8.97 ± 0.69	9.25 ± 0.73	8.75 ± 0.57	<0.001
TG/HDL-C ratio	4.33 ± 2.24	5.32 ± 2.47	3.56 ± 1.71	<0.001
BAR	8.9 ± 4.7	12.4 ± 5.3	6.2 ± 2.4	<0.001
CAR	18.4 (7.1–38.8)	29.8 (14.2–52.4)	9.1 (3.8–21.3)	<0.001
LAR	0.69 (0.41–1.21)	1.02 (0.63–1.74)	0.46 (0.29–0.73)	<0.001
DAR	1.74 (0.81–4.06)	3.12 (1.74–6.31)	0.95 (0.48–1.86)	<0.001
FAR	129 ± 44	159 ± 47	106 ± 29	<0.001
PNI	43.8 ± 7.1	39.4 ± 6.3	47.2 ± 5.4	<0.001

Data are presented as mean ± standard deviation, median (interquartile range), or number (%), as appropriate. CRP: C-reactive protein; HDL-C: high-density lipoprotein cholesterol; BNP: B-type natriuretic peptide; TyG: triglyceride–glucose index; BAR: blood urea nitrogen-to-albumin ratio; CAR: C-reactive protein-to-albumin ratio; LAR: lactate-to-albumin ratio; DAR: D-dimer-to-albumin ratio; FAR: fibrinogen-to-albumin ratio; PNI: prognostic nutritional index; RVD: right ventricular dysfunction.

**Table 4:** Univariable and multivariable logistic regression analyses for factors associated with RVD.

Variable	Univariable OR (95% CI)	*p*-Value	Multivariable OR (95% CI)	*p*-Value
Age (per 1-year increase)	1.03 (1.01–1.05)	0.004	1.02 (0.99–1.04)	0.108
Active malignancy	1.99 (1.10–3.60)	0.023	1.41 (0.69–2.88)	0.342
Heart rate (per 1 bpm increase)	1.05 (1.03–1.07)	<0.001	1.02 (1.00–1.04)	0.028
Oxygen saturation (per 1% increase)	0.89 (0.85–0.93)	<0.001	0.95 (0.91–0.99)	0.014
Main pulmonary artery involvement	4.31 (2.51–7.42)	<0.001	2.08 (1.07–4.04)	0.031
Saddle embolism	5.21 (2.37–11.46)	<0.001	2.29 (0.96–5.48)	0.061
Troponin (per 10 ng/L increase)	1.08 (1.05–1.11)	<0.001	1.04 (1.01–1.07)	0.012
Log_2_-transformed normalized BNP/NT-proBNP, per doubling	1.12 (1.08–1.16)	<0.001	1.07 (1.03–1.11)	<0.001
TyG index	2.84 (1.91–4.22)	<0.001	1.63 (1.01–2.63)	0.045
TG/HDL-C ratio	1.32 (1.18–1.49)	<0.001	1.08 (0.92–1.27)	0.336
BAR	1.29 (1.20–1.38)	<0.001	1.15 (1.05–1.26)	0.003
CAR	1.06 (1.04–1.08)	<0.001	1.03 (1.01–1.06)	0.009
LAR	3.87 (2.43–6.15)	<0.001	2.04 (1.12–3.72)	0.020
DAR	1.42 (1.27–1.58)	<0.001	1.18 (1.04–1.34)	0.011
FAR	1.03 (1.02–1.04)	<0.001	1.01 (1.00–1.02)	0.084
PNI	0.86 (0.82–0.90)	<0.001	0.93 (0.88–0.98)	0.006

OR, odds ratio; CI, confidence interval; BNP, B-type natriuretic peptide; TyG, triglyceride–glucose index; BAR, blood urea nitrogen-to-albumin ratio; CAR, C-reactive protein-to-albumin ratio; LAR, lactate-to-albumin ratio; DAR, D-dimer-to-albumin ratio; FAR, fibrinogen-to-albumin ratio; PNI, prognostic nutritional index; HDL-C, high-density lipoprotein cholesterol. No significant multicollinearity was observed among variables included in the multivariable model (all VIF values < 2.0).

**Table 5:** ROC analysis of composite indices for discriminating between patients with and without RVD.

Variable	AUC (95% CI)	Cut-Off Value	Sensitivity (%)	Specificity (%)	Youden Index	*p*-Value
TyG index	0.742 (0.683–0.801)	>9.02	71.2	68.4	0.396	<0.001
TG/HDL-C ratio	0.721 (0.661–0.781)	>4.28	69.5	65.1	0.346	<0.001
BAR	0.812 (0.760–0.864)	>8.7	77.1	75.7	0.528	<0.001
CAR	0.846 (0.799–0.893)	>16.5	80.5	78.9	0.594	<0.001
LAR	0.823 (0.772–0.874)	>0.84	75.4	79.6	0.550	<0.001
DAR	0.858 (0.812–0.904)	>1.85	82.2	80.3	0.625	<0.001
FAR	0.781 (0.724–0.839)	>132	72.9	73.7	0.466	<0.001
PNI	0.837 (0.788–0.886)	≤42.5	78.8	77.0	0.558	<0.001

AUC, area under the curve; CI, confidence interval; TyG, triglyceride–glucose index; BAR, blood urea nitrogen-to-albumin ratio; CAR, C-reactive protein-to-albumin ratio; LAR, lactate-to-albumin ratio; DAR, D-dimer-to-albumin ratio; FAR, fibrinogen-to-albumin ratio; PNI, prognostic nutritional index.

**Table 6:** Comparison of ROC curves using DeLong analysis.

Comparison	Difference in AUC	Standard Error	Z Statistic	*p*-Value
DAR vs. CAR	0.012	0.018	0.67	0.503
DAR vs. PNI	0.021	0.019	1.11	0.267
DAR vs. LAR	0.035	0.021	1.67	0.095
DAR vs. BAR	0.046	0.022	2.09	0.037
DAR vs. FAR	0.077	0.025	3.08	0.002
DAR vs. TyG	0.116	0.028	4.14	<0.001
DAR vs. TG/HDL-C	0.137	0.030	4.57	<0.001
CAR vs. PNI	0.009	0.017	0.53	0.597
CAR vs. LAR	0.023	0.020	1.15	0.250
CAR vs. BAR	0.034	0.021	1.62	0.105
CAR vs. FAR	0.065	0.024	2.71	0.007
CAR vs. TyG	0.104	0.027	3.85	<0.001
CAR vs. TG/HDL-C	0.125	0.029	4.31	<0.001
PNI vs. LAR	0.014	0.019	0.74	0.459
PNI vs. BAR	0.025	0.020	1.25	0.211
PNI vs. FAR	0.056	0.023	2.43	0.015
PNI vs. TyG	0.095	0.026	3.65	<0.001
PNI vs. TG/HDL-C	0.116	0.028	4.14	<0.001

AUC, area under the curve; DAR, D-dimer-to-albumin ratio; CAR, C-reactive protein-to-albumin ratio; PNI, prognostic nutritional index; LAR, lactate-to-albumin ratio; BAR, blood urea nitrogen-to-albumin ratio; FAR, fibrinogen-to-albumin ratio; TyG, triglyceride–glucose index; TG/HDL-C, triglyceride-to-high-density lipoprotein cholesterol ratio.

**Table 7:** Correlation analysis between composite indices and markers of RVD.

Variable	RV/LV Ratio (CTPA) r	*p*-Value	RV/LV Ratio (ECO) r	*p*-Value	TAPSE r	*p*-Value
TyG index	0.284	<0.001	0.261	<0.001	−0.249	<0.001
TG/HDL-C ratio	0.251	<0.001	0.228	0.001	−0.217	0.002
BAR	0.471	<0.001	0.438	<0.001	−0.422	<0.001
CAR	0.566	<0.001	0.531	<0.001	−0.508	<0.001
LAR	0.518	<0.001	0.489	<0.001	−0.473	<0.001
DAR	0.612	<0.001	0.587	<0.001	−0.551	<0.001
FAR	0.421	<0.001	0.397	<0.001	−0.382	<0.001
PNI	−0.547	<0.001	−0.523	<0.001	0.501	<0.001
Troponin	0.594	<0.001	0.558	<0.001	−0.527	<0.001
BNP/NT-proBNP	0.628	<0.001	0.601	<0.001	−0.573	<0.001

Spearman correlation analysis was performed. CTPA, computed tomography pulmonary angiography; RV, right ventricle; LV, left ventricle; TAPSE, tricuspid annular plane systolic excursion; TyG, triglyceride–glucose index; BAR, blood urea nitrogen-to-albumin ratio; CAR, C-reactive protein-to-albumin ratio; LAR, lactate-to-albumin ratio; DAR, D-dimer-to-albumin ratio; FAR, fibrinogen-to-albumin ratio; PNI, prognostic nutritional index; BNP, B-type natriuretic peptide.

**Table 8:** Management and clinical outcomes according to RVD status.

Variable	Total (n = 270)	RVD (+) (n = 118)	RVD (−) (n = 152)	*p*-Value
Intensive care unit admission, n (%)	121 (44.8)	79 (66.9)	42 (27.6)	<0.001
Length of ICU stay, days	4 (2–8)	6 (3–10)	2 (1–4)	<0.001
Length of hospital stay, days	8 (5–13)	10 (7–15)	7 (4–11)	<0.001
Mechanical ventilation requirement, n (%)	42 (15.6)	31 (26.3)	11 (7.2)	<0.001
Non-invasive ventilation requirement, n (%)	58 (21.5)	36 (30.5)	22 (14.5)	0.002
Vasopressor/inotropic support, n (%)	36 (13.3)	28 (23.7)	8 (5.3)	<0.001
Thrombolytic therapy, n (%)	33 (12.2)	27 (22.9)	6 (3.9)	<0.001
Catheter-directed intervention/embolectomy, n (%)	11 (4.1)	9 (7.6)	2 (1.3)	0.011
Major bleeding, n (%)	14 (5.2)	10 (8.5)	4 (2.6)	0.035
Recurrent pulmonary embolism, n (%)	12 (4.4)	8 (6.8)	4 (2.6)	0.107
Deep vein thrombosis, n (%)	49 (18.1)	29 (24.6)	20 (13.2)	0.018
Hemodynamic deterioration during hospitalization, n (%)	31 (11.5)	24 (20.3)	7 (4.6)	<0.001
Cardiopulmonary arrest, n (%)	15 (5.6)	12 (10.2)	3 (2.0)	0.005
In-hospital mortality, n (%)	27 (10.0)	21 (17.8)	6 (3.9)	<0.001
30-day mortality, n (%)	31 (11.5)	24 (20.3)	7 (4.6)	<0.001

Data are presented as median (interquartile range) or number (%), as appropriate. ICU: intensive care unit; RVD: right ventricular dysfunction.

## Data Availability

The original contributions presented in this study are included in the article. Further inquiries can be directed to the corresponding author.

## Supplementary Materials

The following supporting information can be downloaded at https://www.mdpi.com/article/10.3390/jcdd13080348/s1, Table S1: Sensitivity analysis restricted to non-high-risk PE; Table S2: Adjusted associations stratified by out-of-hospital and in-hospital PE, including interaction *p*-values; Table S3: Alternative multivariable logistic regression model replacing DAR with log_2_-transformed D-dimer.

## Abbreviations

AUC	Area Under the Curve
BAR	Blood Urea Nitrogen-to-Albumin Ratio
BNP	B-type Natriuretic Peptide
CAR	C-Reactive Protein-to-Albumin Ratio
CI	Confidence Interval
CRP	C-Reactive Protein
CTEPH	Chronic Thromboembolic Pulmonary Hypertension
CTPA	Computed Tomography Pulmonary Angiography
DAR	D-dimer-to-Albumin Ratio
DVT	Deep Vein Thrombosis
FAR	Fibrinogen-to-Albumin Ratio
HDL-C	High-Density Lipoprotein Cholesterol
ICU	Intensive Care Unit
IQR	Interquartile Range
LAR	Lactate-to-Albumin Ratio
LV	Left Ventricle
OR	Odds Ratio
PE	Pulmonary Embolism
PNI	Prognostic Nutritional Index
ROC	Receiver Operating Characteristic
RV	Right Ventricle
RVD	Right Ventricular Dysfunction
SD	Standard Deviation
TAPSE	Tricuspid Annular Plane Systolic Excursion
TG/HDL-C	Triglyceride-to-High-Density Lipoprotein Cholesterol Ratio
TyG	Triglyceride–Glucose Index
