# Supplementary material for: Association of Composite Metabolic, Inflammatory, and Nutritional Indices with Right Ventricular Dysfunction in Acute Pulmonary Embolism: A Retrospective Cohort Study
Source: J Cardiovasc Dev Dis. 2026 Jul 24;13(8):348. doi: 10.3390/jcdd13080348 (PMC13513327; doi:10.3390/jcdd13080348)
Supplement: Supplementary file 1 [file jcdd-13-00348-s001.zip › jcdd-4416899-supplementary.pdf]

Supplementary Table S1: Sensitivity analysis restricted to non-high-risk PE.

Composite index	Univariable OR (95% CI)	p-value	Adjusted OR (95% CI)	p-value
TyG index	2.31 (1.52–3.51)	<0.001	1.49 (0.91–2.43)	0.111
TG/HDL-C ratio	1.27 (1.12–1.44)	<0.001	1.06 (0.89–1.26)	0.512
BAR	1.24 (1.15–1.34)	<0.001	1.11 (1.02–1.21)	0.015
CAR	1.05 (1.03–1.07)	<0.001	1.025 (1.005–1.046)	0.014
LAR	3.28 (2.00–5.38)	<0.001	1.86 (1.01–3.42)	0.047
DAR	1.36 (1.21–1.53)	<0.001	1.15 (1.01–1.31)	0.034
FAR	1.026 (1.017–1.036)	<0.001	1.009 (0.998–1.020)	0.108
PNI	0.87 (0.83–0.91)	<0.001	0.94 (0.89–0.99)	0.018

OR: odds ratio; CI: confidence interval; RVD: right ventricular dysfunction; PE: pulmonary embolism; TyG: triglyceride–glucose index; TG/HDL-C: triglyceride-to-high-density lipoprotein cholesterol ratio; BAR: blood urea nitrogen-to-albumin ratio; CAR: C-reactive protein-to-albumin ratio; LAR: lactate-to-albumin ratio; DAR: D-dimer-to-albumin ratio; FAR: fibrinogen-to-albumin ratio; PNI: prognostic nutritional index.

Supplementary Table S2: Adjusted associations stratified by out-of-hospital and in-hospital PE, including interaction p-values.

Composite index	Out-of-hospital PE, adjusted OR (95% CI)	p-value	In-hospital PE, adjusted OR (95% CI)	p-value	p for interaction
TyG index	1.70 (1.02–2.85)	0.043	1.29 (0.63–2.64)	0.486	0.497
TG/HDL-C ratio	1.10 (0.90–1.34)	0.343	1.03 (0.77–1.38)	0.832	0.748
BAR	1.14 (1.04–1.25)	0.005	1.10 (0.99–1.22)	0.073	0.581
CAR	1.032 (1.010–1.055)	0.004	1.025 (1.001–1.050)	0.041	0.673
LAR	2.11 (1.08–4.13)	0.029	1.67 (0.71–3.94)	0.240	0.608
DAR	1.20 (1.04–1.39)	0.013	1.16 (1.00–1.35)	0.049	0.742
FAR	1.011 (0.999–1.023)	0.071	1.006 (0.990–1.022)	0.461	0.592
PNI	0.92 (0.87–0.98)	0.008	0.93 (0.87–0.99)	0.027	0.804

OR: odds ratio; CI: confidence interval; RVD: right ventricular dysfunction; PE: pulmonary embolism; TyG: triglyceride–glucose index; TG/HDL-C: triglyceride-to-high-density lipoprotein cholesterol ratio; BAR: blood urea nitrogen-to-albumin ratio; CAR: C-reactive protein-to-albumin ratio; LAR: lactate-to-albumin ratio; DAR: D-dimer-to-albumin ratio; FAR: fibrinogen-to-albumin ratio; PNI: prognostic nutritional index.

Supplementary Table S3: Alternative multivariable logistic regression model replacing DAR with log_2-transformed D-dimer.

Variable	Adjusted OR (95% CI)	p-value
Age, per 1-year increase	1.02 (0.99–1.04)	0.126
Active malignancy	1.38 (0.67–2.85)	0.384
Heart rate, per 1 beat/min increase	1.02 (1.00–1.04)	0.031
Oxygen saturation, per 1% increase	0.95 (0.91–0.99)	0.017
Main pulmonary artery involvement	2.04 (1.04–4.00)	0.038
Saddle embolism	2.21 (0.92–5.31)	0.076
Troponin, per 10 ng/L increase	1.04 (1.01–1.07)	0.015
Log_2-transformed normalized BNP/NT-proBNP, per doubling	1.07 (1.03–1.11)	<0.001
TyG index	1.59 (0.98–2.58)	0.061
TG/HDL-C ratio	1.07 (0.91–1.26)	0.401
BAR	1.14 (1.04–1.25)	0.005
CAR	1.03 (1.01–1.06)	0.012
LAR	1.96 (1.07–3.61)	0.030
Log_2-transformed D-dimer, per doubling	1.43 (1.15–1.78)	0.001
FAR	1.01 (1.00–1.02)	0.102
PNI	0.94 (0.89–0.98)	0.008

OR: odds ratio; CI: confidence interval; RVD: right ventricular dysfunction; DAR: D-dimer-to-albumin ratio; BNP: B-type natriuretic peptide; TyG: triglyceride–glucose index; TG/HDL-C: triglyceride-to-high-density lipoprotein cholesterol ratio; BAR: blood urea nitrogen-to-albumin ratio; CAR: C-reactive protein-to-albumin ratio; LAR: lactate-to-albumin ratio; FAR: fibrinogen-to-albumin ratio; PNI: prognostic nutritional index.
